# Phosphatase and Tensin Homolog Inhibition in Proteolipid Protein 1-Expressing Cells Stimulates Neurogenesis and Gliogenesis in the Postnatal Enteric Nervous System

**DOI:** 10.3390/biom14030346

**Published:** 2024-03-13

**Authors:** Crystal Woods, Amanda R. Flockton, Jaime Belkind-Gerson

**Affiliations:** 1 Department of Pediatrics, Section of Gastroenterology, Hepatology and Nutrition, University of Colorado, Aurora, CO 80045, USA; crystal.woods@cuanschutz.edu (C.W.); amanda.flockton@cuanschutz.edu (A.R.F.); 2 Neurogastroenterology and Motility Program, Digestive Health Institute, Children’s Hospital Colorado, Aurora, CO 80045, USA

**Keywords:** enteric nervous system, enteric glia, enteric neurons, gastrointestinal motility, Pten, Plp1, Calb2, short-bowel syndrome

## Abstract

Phosphatase and tensin homolog (Pten) is a key regulator of cell proliferation and a potential target to stimulate postnatal enteric neuro- and/or gliogenesis. To investigate this, we generated two tamoxifen-inducible Cre recombinase murine models in which *Pten* was conditionally ablated, (1) in glia (*Plp1*-expressing cells) and (2) in neurons (*Calb2*-expressing cells). Tamoxifen-treated adult (7–12 weeks of age; *n* = 4–15) mice were given DSS to induce colitis, EdU to monitor cell proliferation, and were evaluated at two timepoints: (1) early (3–4 days post-DSS) and (2) late (3–4 weeks post-DSS). We investigated gut motility and evaluated the enteric nervous system. *Pten* inhibition in *Plp1*-expressing cells elicited gliogenesis at baseline and post-DSS (early and late) in the colon, and neurogenesis post-DSS late in the proximal colon. They also exhibited an increased frequency of colonic migrating motor complexes (CMMC) and slower whole gut transit times. *Pten* inhibition in *Calb2*-expressing cells did not induce enteric neuro- or gliogenesis, and no alterations were detected in CMMC or whole gut transit times when compared to the control at baseline or post-DSS (early and late). Our results merit further research into *Pten* modulation where increased glia and/or slower intestinal transit times are desired (e.g., short-bowel syndrome and rapid-transit disorders).

## 1. Introduction

The enteric nervous system (ENS) comprises complex networks of neurons and glia that form the myenteric and submucosal plexuses of the gastrointestinal (GI) tract [1]. Homeostasis of these networks are crucial to the proper functioning of intestinal motor and secretory processes [1]. The occurrence of postnatal ENS injuries are exceedingly common, resulting in distressing symptoms in the patient population (e.g., inflammatory bowel disease). While postnatal ENS regeneration has been reported in response to intestinal inflammation, denervation, antibiotics, and 5HT agonists, the regenerative response of enteric neurons and glia has been limited at best [2,3,4,5,6,7,8]. Understanding the pathways involved in the regulation of enteric neuronal and glial stemness are critical to harnessing the regenerative potential of the ENS and would have significant positive impacts on patient care and quality of life.

Prior studies have implicated phosphatase and tensin homolog (Pten) as a potential target to promote enteric neuronal and/or glial cell proliferation. Pten has an important role in the regulation of mitosis, cell survival, and migration through the suppression of the PI3K/AKT/mTOR pathway [9,10]. As a result, Pten functions as a tumor suppressor and is often found to be inactive in various cancers, where cell proliferation proceeds unchecked [9,10]. In Pten studies focused on the central nervous system (CNS), *Pten* deficient astrocytes exhibited elevated levels of proliferation [11], and Pten suppression via miR-26a overexpression promoted the axonal growth of hippocampal neurons [12]. Of particular interest, Pten expression has been shown to be a critical component for the normal development of the ENS [13,14,15].

Puig et al. conditionally knocked out (cKO) *Pten* in embryonic enteric neuronal cells and this resulted in a lethal mutation, with mice dying 2–3 weeks after birth with symptoms of chronic intestinal pseudoobstruction (i.e., ganglioneuromatosis, hyperplasia, and hypertrophy of enteric neuronal cells) [13]. To study the role Pten plays in the postnatal ENS, researchers have utilized inducible cKO mouse models and Pten inhibitors [16,17]. Becker et al. observed that in an ex vivo model, ENS neurogenesis was enhanced in response to a Pten inhibitor [16]. More recently, Kulkarni et al. ablated *Pten* in *Nestin*-expressing cells in an in vivo model and detected a dramatic increase in postnatal enteric neuronal numbers [17]. Everything considered, these studies suggested a potential strategy for enhancing ENS repair in response to an intestinal injury.

We hypothesized that inhibiting Pten expression in enteric neurons and glia could promote postnatal enteric neuro- and/or gliogenesis in vivo, and this regenerative effect could positively impact intestinal function, especially following an ENS injury. To test this hypothesis, we generated two murine models, one that conditionally eliminated Pten expression in proteolipid protein 1 (*Plp1*)-expressing glia and another in calbindin 2 (*Calb2*)-expressing neurons using a tamoxifen-inducible Cre/*lox* system. *Plp1* was an ideal target, since most enteric glia express *Plp1* [18]. *Calb2*-expressing neurons were of particular interest, since they are inclusive of mostly excitatory neurons, and their subsequent increase in number and/or density could have a positive effect on intestinal motility [19]. We then investigated whether targeted glial and neuronal *Pten* eradication induced postnatal enteric neuro- and/or gliogenesis and evaluated the functional repercussions on the colon and ileum of the cKO models at baseline and after dextran sulfate sodium (DSS)-induced colitis. We looked at two timepoints post-injury: early (3–4 days) and late (3–4 weeks).

## 2. Materials and Methods

### 2.1. Animal Model

Animal procedures were approved by the University of Colorado’s Institutional Animal Care and Use Committee under protocol #00088. Individual mouse strains (Table 1) were obtained from The Jackson Laboratory (Bar Harbor, ME, USA) and crossed to create tamoxifen-inducible *Pten* cKO mice. Transgenic male and female mice homozygous for *Pten^flox^* [20] (*Pten^fl/fl^*) and hemizygous for *Plp1CreER^T^* [21] (*Pten^fl/fl^;Plp1CreER^T^*), homozygous for *Pten^flox^* and hemizygous for *Calb2CreER^T2^* [22] (*Pten^fl/fl^;Calb2CreER^T2^*) and littermate controls that were homozygous for *Pten^flox^* and negative for Cre recombinase were used at 7–12 weeks of age. All mice were administered 5 mg of tamoxifen (#T5648; Sigma-Aldrich; St. Louis, MO, USA) suspended in corn oil (#C8267; Sigma-Aldrich) daily for 5 consecutive days by intraperitoneal (IP) injection (100 μL). In total, 3% DSS (#J14489-22; Thermo Fisher Scientific; Waltham, MA, USA) was provided via drinking water for 7 consecutive days beginning on the last day of tamoxifen injections. Control animals were provided untreated drinking water. Mice were administered 1 mg of 5-ethynyl-2′-deoxyuridine (EdU; #A10044; Life Technologies; Carlsbad, CA, USA) by IP injection on the final day of DSS and 3 days post-treatment end. Animals were euthanized by CO_2_ asphyxiation followed by cervical dislocation 3–4 days post-DSS (early) and 3–4 weeks post-DSS (late).

### 2.2. Ex Vivo Colonic Motility

As previously detailed, we modified Swaminathan et al.’s method to film and evaluate colonic motility ex vivo [23,24]. Briefly, colons were flushed with sterile PBS and submerged in continuously flowing, oxygenated Krebs buffer, maintained at 37 °C in an organ bath. The colons were cannulated at the most proximal (oral) and distal (anal) ends, secured in place with suture and minutien pins, and the cannulas were connected to reservoirs containing oxygenated Krebs buffer, maintained at 2 cm H_2_O pressure and 37 °C. Colons were filmed using the Gastrointestinal Motility Monitor (GIMM) system (Catamount R&D; St. Albans, VT, USA), and the video files were converted into spatiotemporal maps using GIMM software, version 2.0.2.14 [25]. The frequency of colonic migrating motor complexes (CMMC) was determined from the spatiotemporal maps. The maps were opened in ImageJ, version 1.54f (National Institutes of Health, Stapleton, NY, USA), where velocity and percent length propagation measurements were made with the GIMM processor plugin. Frequency and velocity measurements were taken from CMMC that propagated at least 50% of the length of the colon. The investigators completing the CMMC measurements were blinded to the genotype and treatment group of the mice.

### 2.3. Whole Gut Transit Time

As previously described, mice were gavaged with 200 μL of 5% Evans blue (#E2129; Sigma-Aldrich, St. Louis, MO, USA), suspended in 5% gum Arabic (#G9752, Sigma-Aldrich) [24]. They were individually housed and fasted 1 h prior to and post-gavage. The time between the gavage to the passage of the first blue fecal pellet was designated as the whole gut transit time.

### 2.4. Paraffin Embedded Colonic Sections

The entire colon was flushed with ice-cold PBS. While submerged in cold PBS, colons were cut longitudinally along the mesenteric border, carefully rolled with a toothpick, and placed in a Histosette I embedding cassette (#M490-9; Simport Scientific, Saint-Mathieu-de-Beloeil, QC, Canada). Cassettes were submerged in 4% formaldehyde (#BP531; Thermo Fisher Scientific) diluted in PBS, and samples were fixed for 24–48 h at 4 °C. Samples were paraffin embedded and sectioned by Rubin Tudor’s Histology Core at the University of Colorado.

#### 2.4.1. Damage Scores

Colon sections were stained with an H&E stain kit (#ab245880; Abcam; Cambridge, UK) according to the manufacturer’s instructions. Sections were evaluated for microscopic damage where each colon section was accessed for goblet cell depletion (absent to extensive, scoring 0–2, respectively), crypt abscesses (absent to extensive, scoring 0–2), muscle thickening (normal to extensive, scoring 0–2), mucosal architectural damage (normal to extensive, scoring 0–2), and inflammatory cellular infiltration (eosinophils, macrophages, and neutrophils, normal to transmural, scoring 0–2) with a cumulative maximum damage score of 10.

#### 2.4.2. Immunofluorescence

As previously detailed, sections were deparaffinized in xylenes (#X5-1; Thermo Fisher Scientific) and rehydrated in a graded ethanol series [26]. Antigen retrieval was performed with 10 mM sodium citrate pH 6 in a pressure cooker on high for 5 min. Sections were washed in PBS then incubated in blocking buffer (10% donkey serum, 10% bovine serum albumin and 1% triton X-100 diluted in PBS) for 1 h at room temperature. Primary antibodies (Table 2) were diluted in blocking buffer and sections were incubated overnight at 4 °C. Slides were washed in PBS and incubated for 1 h in secondary antibodies (Table 2) diluted with blocking buffer. Slides were washed with PBS then counterstained with DAPI (#D3571; Invitrogen, San Diego, CA, USA). For EdU visualization, a Click-IT Plus EdU Alexa Fluor 488 Imaging Kit (#C10637; Invitrogen) was used according to the manufacturer’s instructions. Prolong Gold (#P36930; Molecular Probes; Eugene, OR, USA) was used to coverslip sections. 10–12, 10× images were taken throughout the colon cross sections. Positive cells were manually counted in each image, averaged, and expressed as positive cells per ganglia.

### 2.5. Wholemount Preparation

As previously described, the entire colon and 5–8 cm of the distal ileum were flushed with ice-cold PBS, submerged in pre-chilled 1 μM nifedipine (#N7634; Sigma-Aldrich) for 10 min, and while submerged, were cut along the mesenteric edge [24]. Tissues were pinned flat in pre-chilled sylgard-coated petri dishes while submerged in PBS. PBS was replaced with 4% formaldehyde and tissues were fixed for 1 h on ice. Tissues were stored in PBS at 4 °C. For myenteric and submucosal plexus wholemount preparations, the muscularis containing the myenteric plexus was gently peeled from the submucosal plexus/mucosal layers prior to staining and imaging.

#### 2.5.1. Immunofluorescence

Wholemount preparations were submerged in primary antibody (Table 2) diluted with 20% dimethyl sulfoxide (#276855, Sigma-Aldrich) in blocking buffer (10% donkey serum, 10% bovine serum albumin, and 1% triton X-100 diluted in PBS) and incubated at 4 °C with gentle rocking for 6 days. The antibody was removed, and tissues were washed with PBS for 1–2 h with gentle rocking at room temperature. Tissues were then incubated in secondary antibodies (Table 2) diluted in blocking buffer for 3 h, then washed with PBS for 2–3 h with gentle rocking, counterstained with DAPI, mounted flat, and cover slipped with Prolong Gold.

#### 2.5.2. Image Analysis

A minimum of 10 ganglia containing at least 5 neurons each were selected in the myenteric and submucosal plexus in 1 cm segments of the distal ileum and proximal colon stained for Sox2 and Hu. Positive cells for the marker(s) present were manually counted, averaged, and expressed as positive cells per ganglia.

### 2.6. Imaging

Images were taken on an Olympus IX83 P27F motorized inverted microscope outfitted with UPSLSAPO 10× and 20× air objectives, and a DP80 camera (Olympus; Shinjuku City, Tokyo, Japan). The investigators completing the imaging, damage scoring, and manual cell counts were blinded to the genotype and treatment group of the mice.

### 2.7. Statistical Analysis

Data were graphed and analyzed using an unpaired *t* test or one-way ANOVA (where indicated) using GraphPad Prism software, version 10.1.1 (GraphPad; San Diego, CA, USA). Individual data points represent individual mice unless noted otherwise. Data were expressed as mean ± standard error (SE). Statistical significance was defined as *p* ≤ 0.05.

## 3. Results

### 3.1. Mice Lacking Pten in Plp1-Expressing Cells Were Not Able to Recover Weight Lost Due to DSS Treatment during the Recovery Period

**Figure 1:**
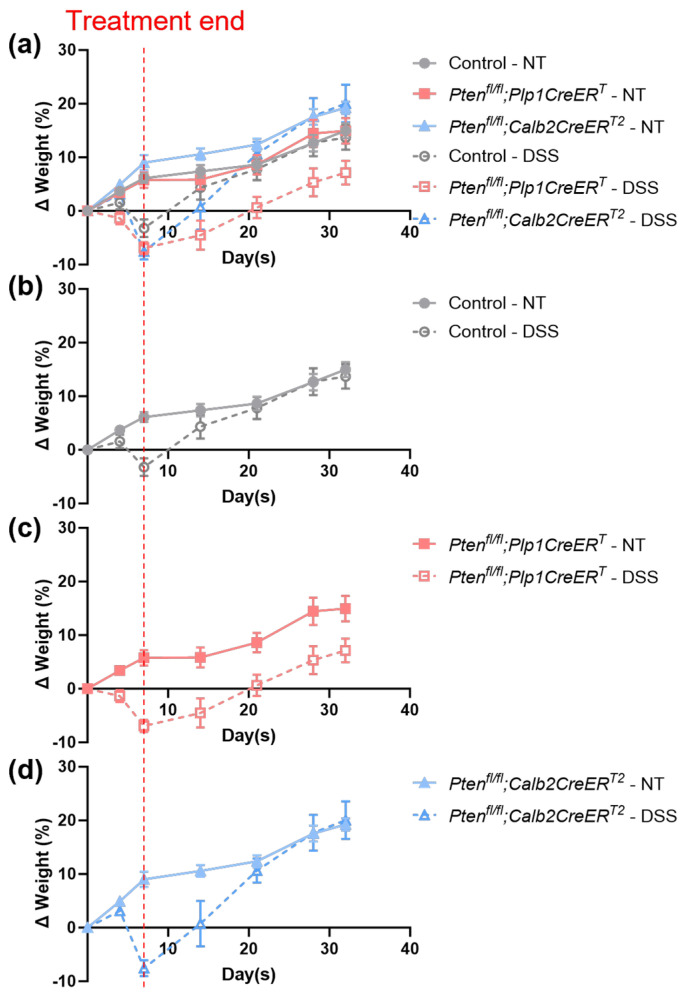
Body weights did not fully recover in DSS-treated mice that lacked *Pten* in *Plp1*-expressing cells. The percent average change in body weights was graphed over time in control (**a**,**b**), *Pten^fl/fl^;Plp1CreER^T^* (**a**,**c**), and *Pten^fl/fl^;Calb2CreER^T2^* (**a**,**d**) mice in groups that received no treatment (solid lines) and DSS (dashed lines). Initial body weights were taken on day 0, before treatment was administered. The treatment endpoint is represented by the vertical red dotted line on day 7. Data was expressed as mean ± SE; NT, no treatment.

To monitor treatment progression, mice were weighed periodically throughout the study. All untreated mice gained weight over time until they reached their endpoint (Figure 1). Mice that were treated with DSS initially lost weight during the treatment but then began gaining weight during the recovery period (Figure 1). The DSS-treated control and *Pten^fl/fl^;Calb2CreER^T2^* mice were able to catch up to the untreated mice by day 21 (Figure 1b,d); however, the *Pten^fl/fl^;Plp1CreER^T^* mice were not able to regain baseline body weight levels (Figure 1c).

**Figure 2:**
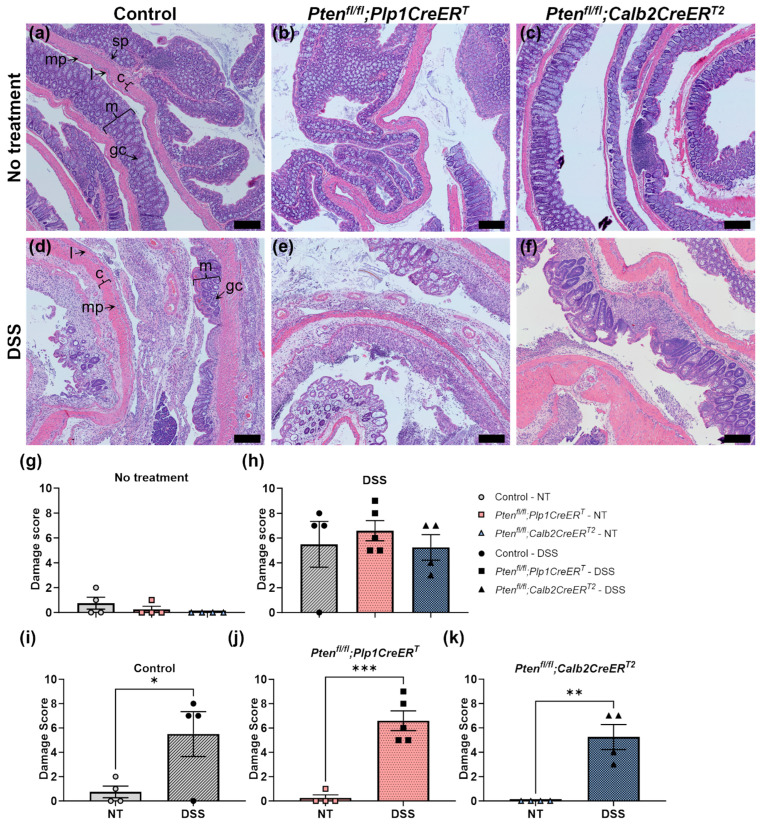
Acute colonic damage was evident in control, *Pten^fl/fl^;Plp1CreER^T^* and *Pten^fl/fl^;Calb2CreER^T2^* mice immediately after DSS treatment but was unaltered between genotypes. Representative images from H&E-stained colon sections from control (**a**,**d**), *Pten^fl/fl^;Plp1CreER^T^* (**b**,**e**), and *Pten^fl/fl^;Calb2CreER^T2^* (**c**,**f**) mice that received no treatment (**a**–**c**), and 2–3 days post-DSS (**d**–**f**). H&E-stained colon sections were assessed for goblet cell depletion (absent to extensive, scoring 0–2, respectively), crypt abscesses (absent to extensive, 0–2), muscle thickening (normal to extensive, 0–2), mucosal architectural damage (normal to extensive; 0–2), and inflammatory cellular infiltration (normal to transmural, 0–2) with a cumulative maximum damage score of 10. Damage scores in mice that received no treatment (**g**) and DSS (**h**), comparing control (**i**), *Pten^fl/fl^;Plp1CreER^T^* (**j**) and *Pten^fl/fl^;Calb2CreER^T2^* (**k**) mice. Each data point represents an individual mouse (*n* = 4, control; *n* = 4–5, *Pten^fl/fl^;Plp1CreER^T^*; *n* = 4, *Pten^fl/fl^;Calb2CreER^T2^*); data were expressed as mean ± SE; * *p* ≤ 0.05, ** *p* ≤ 0.01, *** *p* ≤ 0.001; one-way ANOVA (**g**,**h**) or unpaired two-tailed *t* test (**i**–**k**); NT, no treatment; c, circular muscle; gc, goblet cell; l, longitudinal muscle; m, mucosa; mp, myenteric plexus; sp, submucosal plexus; scale bar, 200 µm.

**Figure 3:**
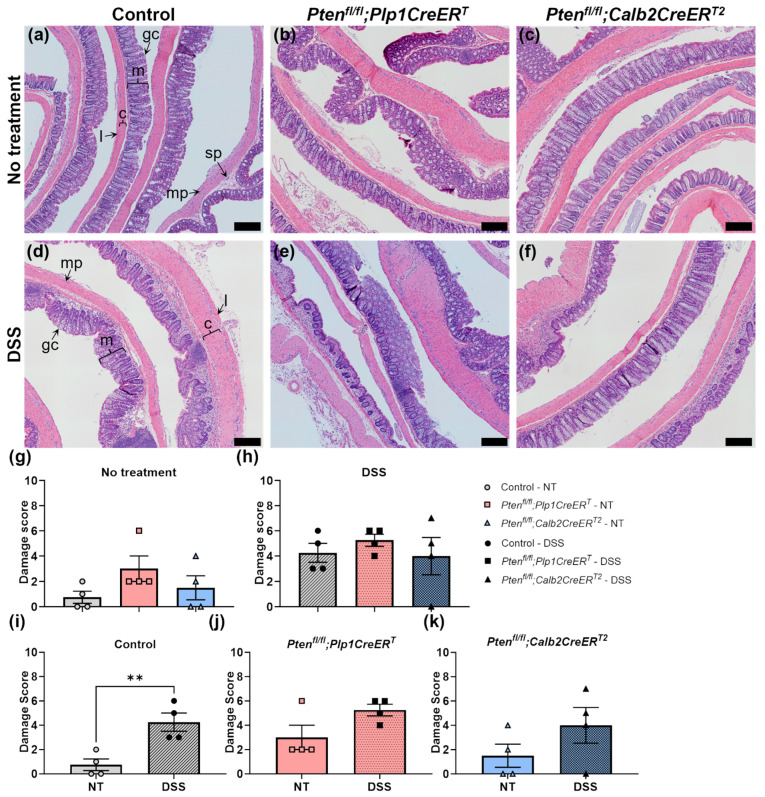
3–4 weeks after DSS treatment, colonic damage was reduced but still present in control, *Pten^fl/fl^;Plp1CreER^T^* and *Pten^fl/fl^;Calb2CreER^T2^* mice but was unaltered between genotype and by treatment in *Pten^fl/fl^;Plp1CreER^T^* and *Pten^fl/fl^;Calb2CreER^T2^* mice. Representative images from H&E-stained colon sections from control (**a**,**d**), *Pten^fl/fl^;Plp1CreER^T^* (**b**,**e**) and *Pten^fl/fl^;Calb2CreER^T2^* (**c**,**f**) in groups that received no treatment (**a**–**c**) and 3–4 weeks post-DSS (**d**–**f**). H&E-stained colon sections were assessed for goblet cell depletion (absent to extensive, scoring 0–2, respectively), crypt abscesses (absent to extensive, 0–2), muscle thickening (normal to extensive, 0–2), mucosal architectural damage (normal to extensive, 0–2), and inflammatory cellular infiltration (normal to transmural, 0–2) with a cumulative maximum damage score of 10. Damage scores in mice that received no treatment (**g**) and 3–4 weeks post-DSS (**h**), comparing control (**i**), *Pten^fl/fl^;Plp1CreER^T^* (**j**) and *Pten^fl/fl^;Calb2CreER^T2^* (**k**) mice. Each data point represents an individual mouse (*n* = 4, control; *n* = 4, *Pten^fl/fl^;Plp1CreER^T^*; *n* = 4, *Pten^fl/fl^;Calb2CreER^T2^*); data were expressed as mean ± SE; ** *p* ≤ 0.01 using one-way ANOVA (**g**,**h**) or unpaired two-tailed *t* test (**i**–**k**);. NT, no treatment; c, circular muscle; gc, goblet cell; l, longitudinal muscle; m, mucosa; mp, myenteric plexus; sp, submucosal plexus; scale bar, 200 µm.

A microscopic assessment of H&E-stained colon sections was completed to evaluate the extent of damage to the colon. Damage scoring confirmed that the DSS treatment regimen induced colitis (Figure 2). There were no significant differences detected at baseline (Figure 2g) or after DSS (Figure 2h) treatment between the control, *Pten^fl/fl^;Plp1CreER^T^* and *Pten^fl/fl^;Calb2CreER^T2^*. All groups exhibited significantly higher levels of damage immediately after DSS treatment (Figure 2i–k). When they were allowed a recovery period, intestinal damage was reduced, but at 4 weeks out, it was not absent in all groups post-DSS (Figure 3).

### 3.2. In Mice Lacking Pten in Plp1-Expressing Cells, the Rate of Colonic Migrating Motor Complexes Was Slightly Elevated during the Recovery Period after DSS Treatment and Intestinal Transit Times Were Slowed When Compared to DSS-Treated Control and Pten^fl/fl^;Calb2CreER^T2^ Mice

**Figure 4:**
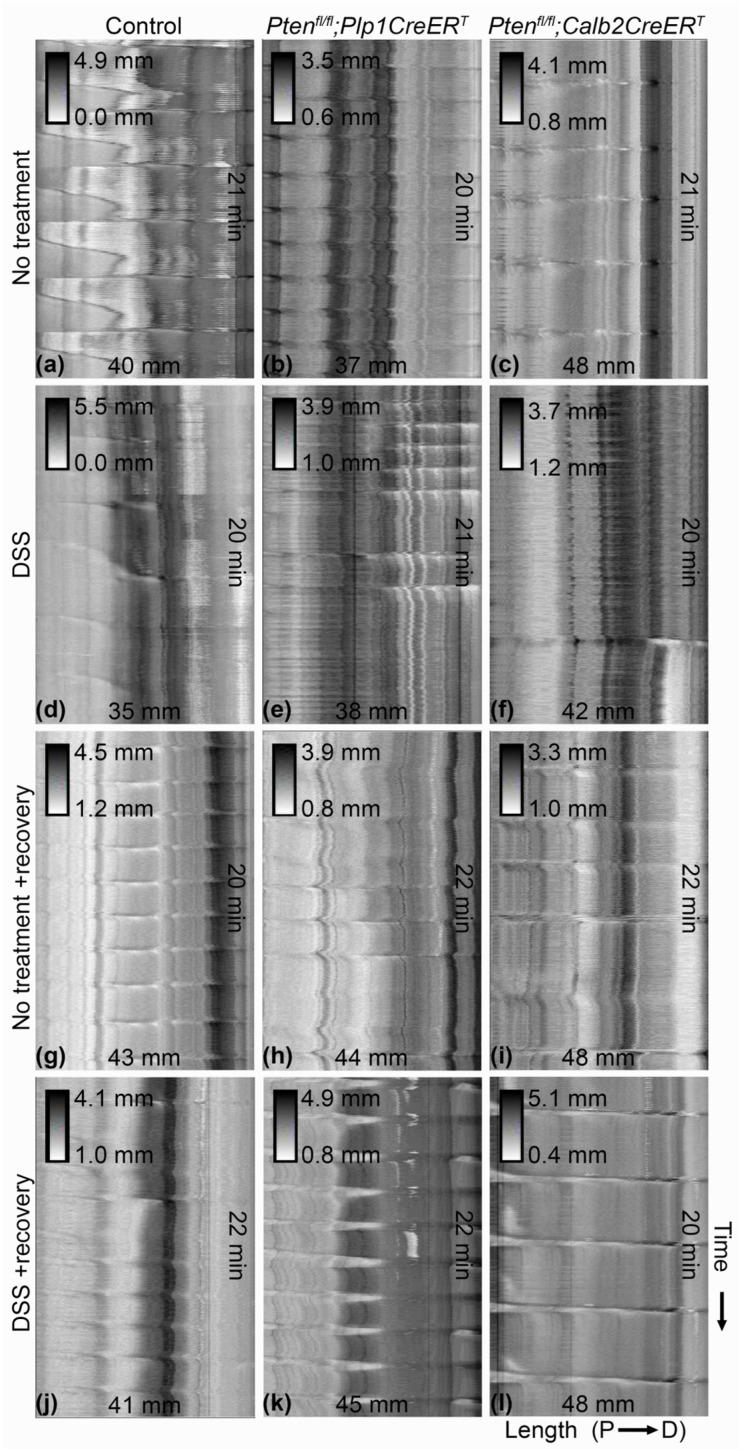
CMMC frequency is slower in control, *Pten^fl/fl^;Plp1CreER^T^* and *Pten^fl/fl^;Calb2CreER^T2^* mice immediately after DSS. Representative spatiotemporal maps of colons from control (**a**,**d**,**g**,**j**), *Pten^fl/fl^;Plp1CreER^T^* (**b**,**e**,**h**,**k**) to *Pten^fl/fl^;Calb2CreER^T2^* (**c**,**f**,**i**,**l**) mice that were untreated (**a**–**c**), DSS-treated (**d**–**f**), and untreated (**g**–**i**) and DSS-treated (**j**–**l**) with a 3–4 week recovery period. Colon length runs horizontally from left (proximal) to right (distal) and time runs vertically, representing approximately 20 min. Shaded insets represent minimum and maximum diameters. CMMC, colonic migrating motor complex; D, distal; P, proximal.

**Figure 5:**
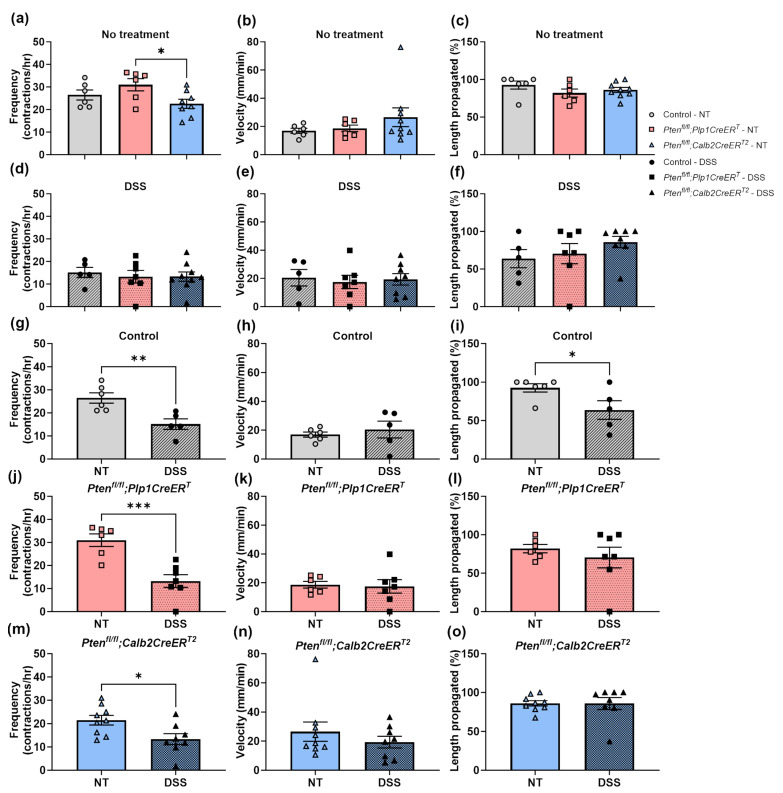
At the early timepoint, CMMC frequency is slower in DSS-treated control, *Pten^fl/fl^;Plp1CreER^T^* and *Pten^fl/fl^;Calb2CreER^T2^* mice and CMMC propagation length is shortened in control mice 3–4 days post-DSS treatment. Spatiotemporal maps of colons from control, *Pten^fl/fl^;Plp1CreER^T^* and *Pten^fl/fl^;Calb2CreER^T2^* mice that had received no treatment or 3–4 days post-DSS were filmed in an organ bath ex vivo and were evaluated for CMMC frequency (**a**,**d**,**g**,**j**,**m**), speed (**b**,**e**,**h**,**k**,**n**), and percent length propagation (**c**,**f**,**i**,**l**,**o**). Each data point represents an individual mouse (*n* = 5–6, control; *n* = 6–7, *Pten^fl/fl^;Plp1CreER^T^*; *n* = 8–9, *Pten^fl/fl^;Calb2CreER^T2^*); data were expressed as mean ± SE; * *p* ≤ 0.05, ** *p* ≤ 0.01, *** *p* ≤ 0.001, using one-way ANOVA (**a**–**f**) or unpaired two-tailed *t* test (**g**–**o**). CMMC, colonic migrating motor complex; NT, no treatment.

**Figure 6:**
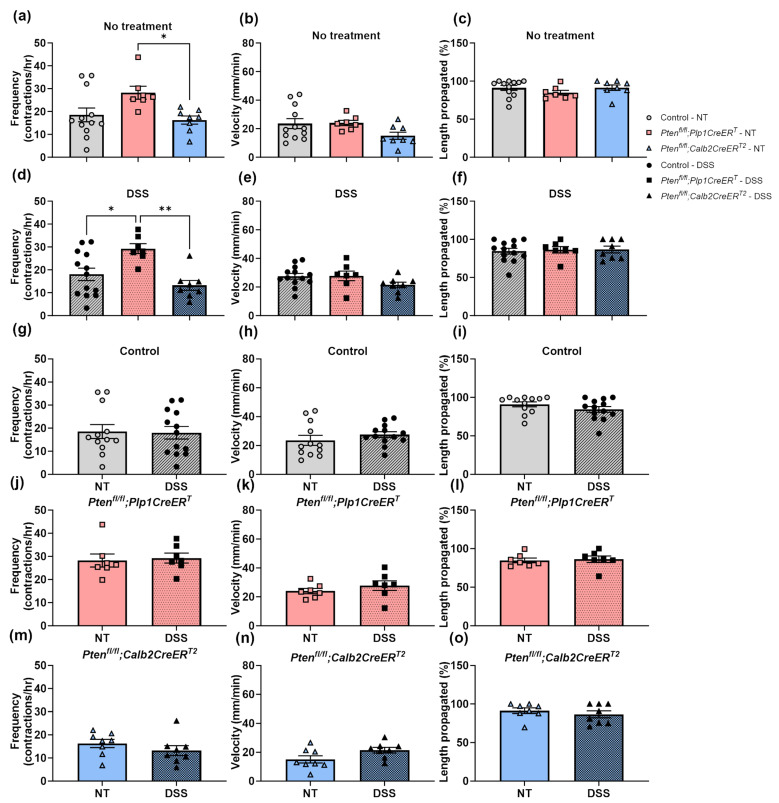
CMMC frequency is slower in *Pten^fl/fl^;Plp1CreER^T^* mice at baseline and 3–4 weeks after DSS treatment. Spatiotemporal maps of colons from control, *Pten^fl/fl^;Plp1CreER^T^* and *Pten^fl/fl^;Calb2CreER^T2^* mice that had received no treatment or 3–4 weeks post-DSS were filmed in an organ bath ex vivo and were evaluated for CMMC frequency (**a**,**d**,**g**,**j**,**m**), speed (**b**,**e**,**h**,**k**,**n**), and percent length propagation (**c**,**f**,**i**,**l**,**o**). Each data point represents an individual mouse (*n* = 12–13, control; *n* = 7, *Pten^fl/fl^;Plp1CreER^T^*; *n* = 8, *Pten^fl/fl^;Calb2CreER^T2^*); data were expressed as mean ± SE; * *p* ≤ 0.05, ** *p* ≤ 0.01 using one-way ANOVA (**a**–**f**) or unpaired two-tailed *t* test (**g**–**o**). CMMC, colonic migrating motor complex; NT, no treatment.

**Figure 7:**
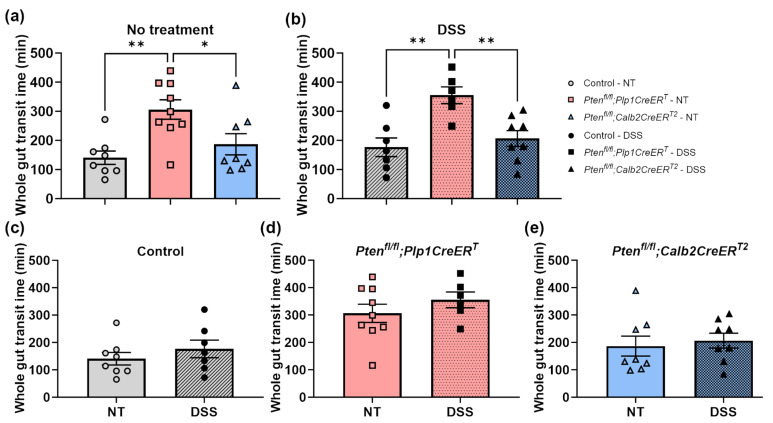
*Pten^fl/fl^;Plp1CreER^T^* had slower transit times when compared to control and *Pten^fl/fl^;Calb2CreER^T2^* mice at baseline (**a**) and 3–4 weeks after DSS (**b**). Whole gut transit times were normalized 3–4 weeks post-DSS treatment in control (**c**), *Pten^fl/fl^;Plp1CreER^T^* (**d**), and *Pten^fl/fl^;Calb2CreER^T2^* (**e**) mice. Each data point represents an individual mouse (*n* = 8–7, control; *n* = 6–9, *Pten^fl/fl^;Plp1CreER^T^*; *n* = 8, *Pten^fl/fl^;Calb2CreER^T2^*); data were expressed as mean ± SE; * *p* ≤ 0.05, ** *p* ≤ 0.01 using one-way ANOVA (**a**,**b**) or unpaired two-tailed *t* test (**c**–**e**). NT, no treatment.

We evaluated the colonic motility (Figure 4, Figure 5 and Figure 6) ex vivo and measured whole gut transit times (Figure 7) in vivo. As expected, all mice that underwent DSS treatment had significantly reduced CMMC frequencies at the early timepoint (Figure 4d–f and Figure 5g,j,m) and, interestingly, only the control mice exhibited incomplete CMMC propagation (Figure 4d and Figure 5i). By the late timepoint, all DSS-treated mice recovered CMMC frequencies to baseline levels (Figure 6g,j,m) and CMMC propagation length returned to normal in the control group (Figure 6i). At baseline, while *Pten^fl/fl^;Plp1CreER^T^* mice appeared to have slightly elevated CMMC frequencies, it was not significantly different from the control mice (Figure 5a and Figure 6a); however, mice treated with DSS and allowed a lengthy recovery, did exhibit significantly increased CMMC frequencies (Figure 6d). CMMC speed was not altered in any treatment group, early or late (Figure 5b,e,h,k,n and Figure 6b,e,h,k,n). Whole gut transit times were significantly slower in the *Pten^fl/fl^;Plp1CreER^T^* group at baseline and after DSS when compared to control and *Pten^fl/fl^;Calb2CreER^T2^* mice (Figure 7).

### 3.3. Immunofluorescent Assessment of the ENS Revealed That Pten Inhibition in Plp1+ Glial Cells Induced Gliogenesis in the Colon at Baseline and after DSS Treatment, and Neurogenesis in the Proximal Colon 3–4 Weeks Post-DSS

**Figure 8:**
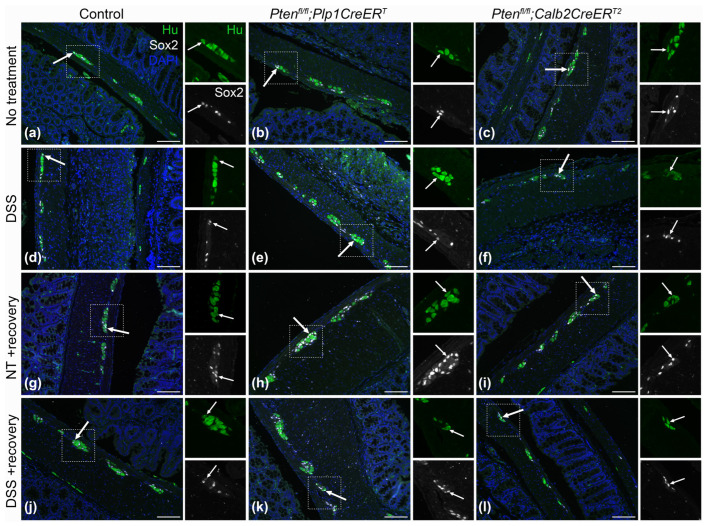
Representative images of Hu (green) and Sox2 (white) immunofluorescence on colon sections from control (**a**,**d**,**g**,**j**), *Pten^fl/fl^;Plp1CreER^T^* (**b**,**e**,**h**,**k**) and *Pten^fl/fl^;Calb2CreER^T2^* (**c**,**f**,**i**,**l**) mice that received no treatment (**a**–**c**) and 3–4 days post-DSS (**d**–**f**), and no treatment (**g**–**i**) and 3–4 weeks post-DSS (**j**–**l**). Magnified ganglia are indicated with a white square, double labelling of Hu and Sox2 are indicated with arrows; NT, no treatment; scale bar, 100 µm.

**Figure 9:**
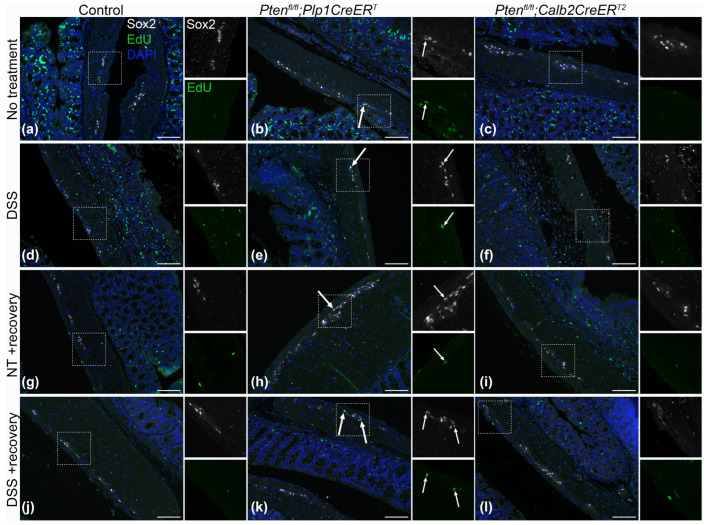
Representative images of Sox2 (white) and EdU (green) immunofluorescence on colon sections from control (**a**,**d**,**g**,**j**), *Pten^fl/fl^;Plp1CreER^T^* (**b**,**e**,**h**,**k**) and *Pten^fl/fl^;Calb2CreER^T2^* (**c**,**f**,**i**,**l**) mice that received no treatment (**a**–**c**) and 3–4 days post-DSS (**d**–**f**), and no treatment (**g**–**i**) and 3–4 weeks post-DSS (**j**–**l**). Magnified ganglia are indicated with a white square, double labelling of Sox2 and EdU are indicated using arrows. EdU, 5-ethynyl-2′-deoxyuridine; NT, no treatment; scale bar, 100 µm.

**Figure 10:**
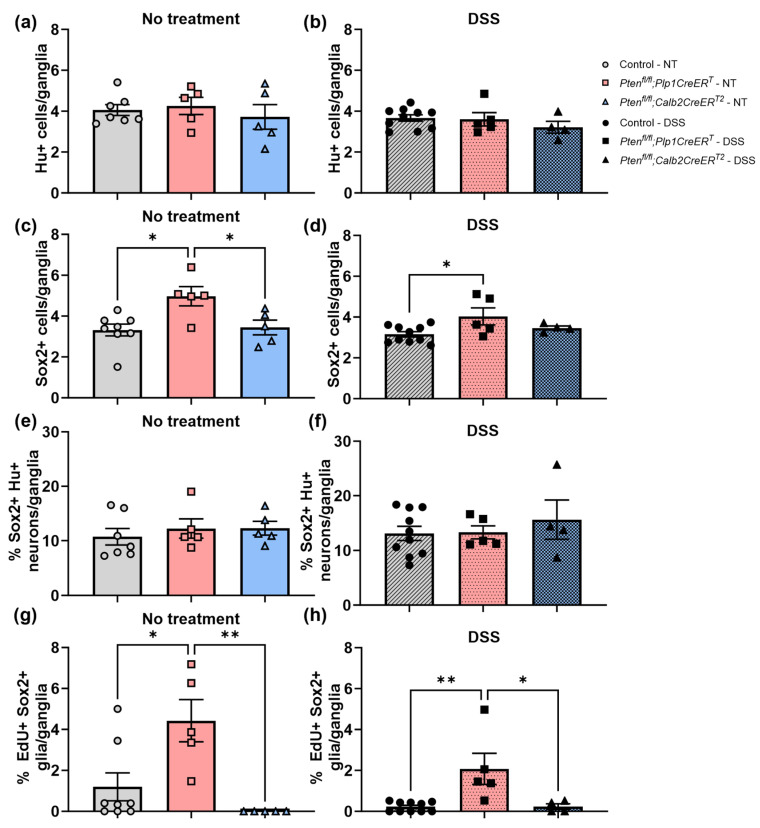
The colons of mice lacking *Pten* in *Plp1*-expressing cells exhibited increased levels of myenteric Sox2+ glial cells and increased numbers of proliferating Sox2+ glial cells at baseline and 3–4 days post-DSS. Hu+ neurons (**a**,**b**), Sox2+ glial cells (**c**,**d**), the percentage of Sox2+ Hu+ neurons (**e**,**f**), and the percentage of EdU+ Sox2+ glial cells (**g**,**h**) per ganglia were counted in colon sections from control, *Pten^fl/fl^;Plp1CreER^T^* and *Pten^fl/fl^;Calb2CreER^T2^* mice that were stained for Hu and Sox2 or Sox2 and EdU. Each data point represents an individual mouse (*n* = 7–10, control; *n* = 5, *Pten^fl/fl^;Plp1CreER^T^*; *n* = 4–5, *Pten^fl/fl^;Calb2CreER^T2^*); data were expressed as mean ± SE. * *p* ≤ 0.05, ** *p* ≤ 0.01; one-way ANOVA; EdU, 5-ethynyl-2′-deoxyuridine; NT, no treatment.

**Figure 11:**
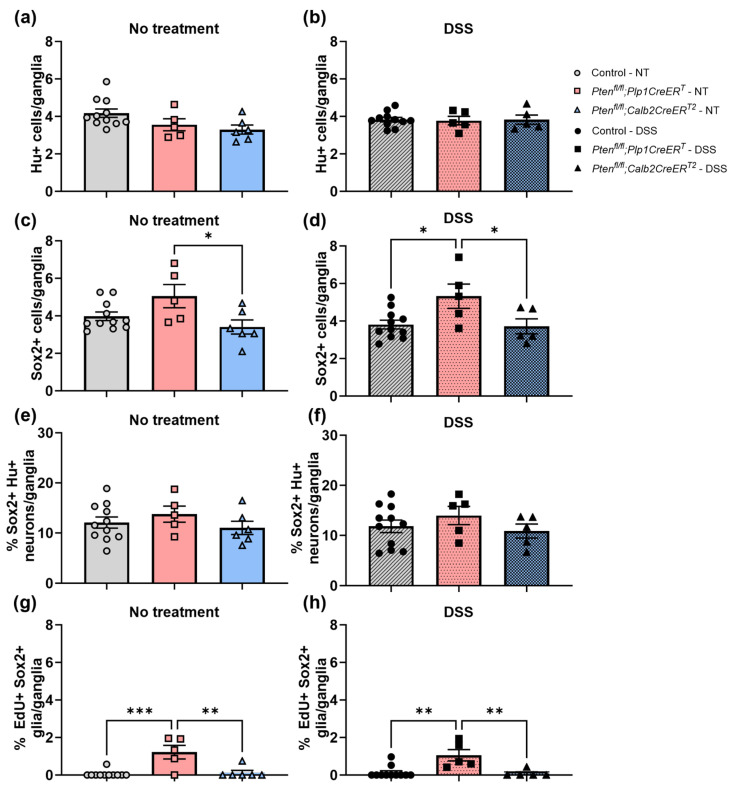
The colons of mice lacking *Pten* in *Plp1*-expressing cells exhibited increased levels of myenteric Sox2+ glial cells and increased numbers of proliferating Sox2+ glial cells at baseline and 3–4 weeks post-DSS. Hu+ neurons (**a**,**b**), Sox2+ glial cells (**c**,**d**), the percentage of Sox2+Hu+ neurons (**e**,**f**), and the percentage of EdU+Sox2+ glial cells (**g**,**h**) per ganglia were counted in colon sections from control, *Pten^fl/fl^;Plp1CreER^T^* and *Pten^fl/fl^;Calb2CreER^T2^* mice that were stained for Hu and Sox2 or Sox2 and EdU. Each data point represents an individual mouse (*n* = 11, control; *n* = 5, *Pten^fl/fl^;Plp1CreER^T^*; *n* = 5–6, *Pten^fl/fl^;Calb2CreER^T2^*); data were expressed as mean ± SE. * *p* ≤ 0.05, ** *p* ≤ 0.01, *** *p* ≤ 0.001; one-way ANOVA; EdU, 5-ethynyl-2′-deoxyuridine; NT, no treatment.

**Figure 12:**
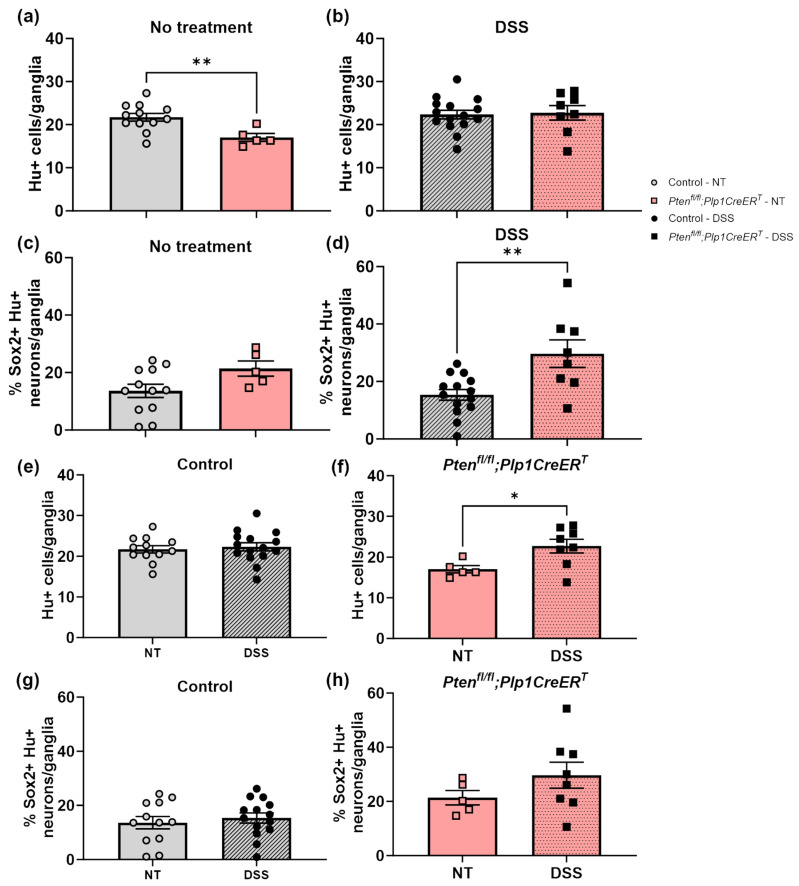
The proximal colon of *Pten^fl/fl^;Plp1CreER^T^* mice had reduced myenteric neuronal numbers at baseline when compared to control but increased neuronal numbers 3–4 weeks after DSS and increased levels of Sox2+Hu+ neurons 3–4 weeks post-DSS treatment. Hu+ neurons (**a**,**b**,**e**,**f**) and the percentage of Sox2+ Hu+ neurons (**c**,**d**,**g**,**h**) per ganglia were counted manually in the myenteric plexus of whole mount preparations of the proximal colon from control (**e**,**g**) and *Pten^fl/fl^;Plp1CreER^T^* (**g**,**h**) mice that were untreated or allowed to recover 3–4 weeks post-DSS treatment. Each data point represents an individual mouse (*n* = 12–15, control; *n* = 5–8, *Pten^fl/fl^;Plp1CreER^T^*); data were expressed as mean ± SE. * *p* ≤ 0.05, ** *p* ≤ 0.01; unpaired two-tailed *t* test; NT, no treatment.

**Figure 13:**
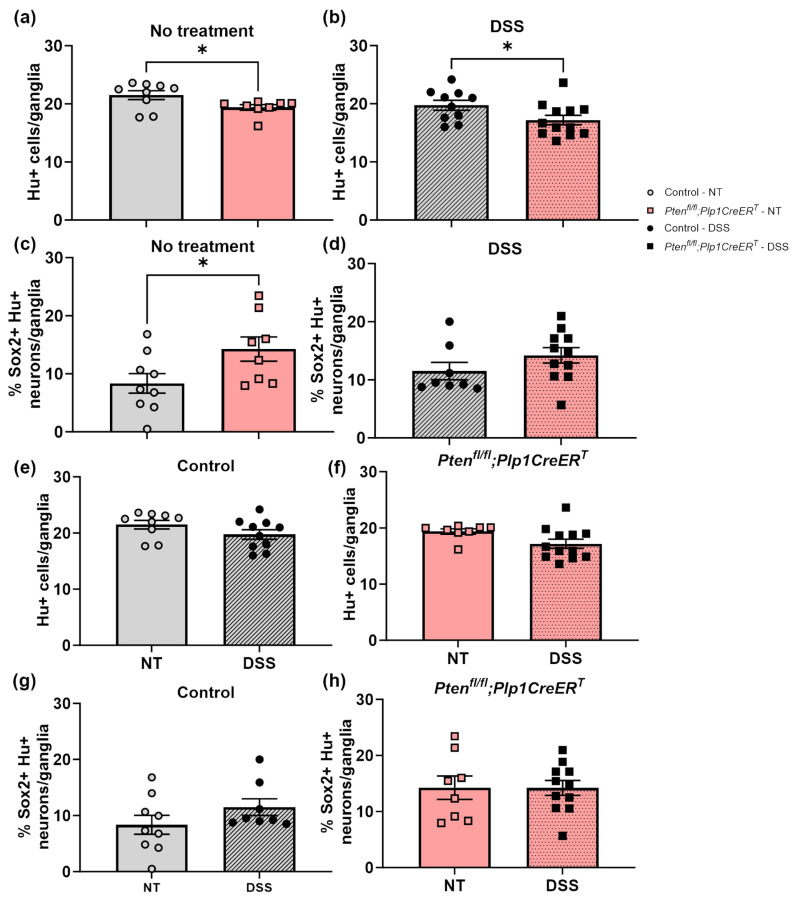
The distal ileum of *Pten^fl/fl^;Plp1CreER^T^* mice had slightly reduced myenteric neuronal numbers at baseline and 3–4 weeks after DSS when compared to control but increased levels of Sox2+Hu+ neurons at baseline. Hu+ neurons (**a**,**b**,**e**,**f**) and the percentage of Sox2+Hu+ neurons (**c**,**d**,**g**,**h**) per ganglia were counted manually in the myenteric plexus of whole mount preparations of the proximal colon from control (**e**,**g**) and *Pten^fl/fl^;Plp1CreER^T^* (**g**,**h**) mice that were untreated or allowed to recover 3–4 weeks post-DSS treatment. Each data point represents an individual mouse (*n* = 8–10, control; *n* = 8–12, *Pten^fl/fl^;Plp1CreER^T^*); data were expressed as mean ± SE. * *p* ≤ 0.05; unpaired two-tailed *t* test; NT, no treatment.

To identify neurons, we used a pan-neuronal marker, Hu (also known as ANNA-1), Sox2 to identify glia, and EdU incorporation to detect cell proliferation. We chose to concentrate on Sox2+ glia due to its importance in ENS regeneration [7,27]. Sox2+Hu+ neurons are known indicators of glial-to-neuronal transdifferentiation and a presumed source of enteric neurogenesis [7,27]. *Pten^fl/fl^;Plp1CreER^T^* colon sections exhibited an increase in Sox2+ myenteric glia and EdU+Sox2+ myenteric glia at baseline and after DSS early and late (Figure 8, Figure 9, Figure 10 and Figure 11); however, somewhat unexpectedly, no overall change was detected in myenteric neuronal numbers of colon sections from either *Pten* cKO at baseline or after DSS at either timepoint (Figure 8, Figure 10 and Figure 11). Since neuronal density can vary throughout the colon [28], subtle differences can be lost; thus, we decided to further investigate possible neurogenesis in the proximal colons and distal ileums of *Pten^fl/fl^;Plp1CreER^T^* and control mice that had been allowed to recover 3–4 weeks post-DSS treatment. *Pten^fl/fl^;Plp1CreER^T^* showed a decrease in Hu+ myenteric neurons at baseline in the proximal colon and distal ileum (Figure 12 and Figure 13). Neuronal numbers returned to control levels post-DSS late in the proximal colon (Figure 12). Additionally, the *Pten^fl/fl^;Plp1CreER^T^* proximal colons (Figure 12d) as well as the distal ileums (Figure 13c) had a significant increase in Sox2+Hu+ neurons. These results suggest that neurogenesis in this model is selective for particular neuronal subsets, such as those that arise from Sox2-expressing glia or progenitors.

## 4. Discussion

We and others have identified ENS renewal and regeneration that persists into postnatal life [7,29,30,31,32]. While numerous studies have demonstrated postnatal enteric neurogenesis in response to an intestinal insult [2,3,4,5,7,8], eliciting and detecting postnatal enteric neurogenesis consistently in rodent models has proven arduous [33,34]. In the present work, we opted to selectively eliminate *Pten* expression in *Plp1*- and *Calb2*-expressing cells. The eradication of *Pten* expression in the ENS was previously reported to stimulate neuro- and gliogenesis; under this assumption, we studied the effects on the ENS and gut motility in response to a commonly used injury model, DSS-induced colitis. Our results demonstrate that selective *Pten* inhibition in *Plp1*-expressing cells has the capacity to alter the ENS and intestinal motility.

Our observations in the ENS using *Pten* cKO models is consistent with Fraser et al. using a similar model in the CNS [11]. Here, we showed that *Pten* deficient glia exhibited elevated enteric glial cell numbers and proliferation throughout the myenteric plexus of the colon (Figure 10c,d,g,h and Figure 11c,d,g,h) and *Pten* deficient neurons did not show any indication of neurogenesis (Figure 10a,b,e,f and Figure 11a,b,e,f).

Despite increased glial proliferation found throughout the myenteric plexus of the colon and indicators of neurogenesis or glial-to-neuronal cell trans-differentiation present post-DSS in the proximal colon, GI function and recovery speed in response to DSS-colitis was not improved in *Pten^fl/fl^;Plp1CreER^T^* mice. In fact, intestinal transit times (Figure 7) were significantly slower and CMMC (Figure 5a and Figure 6a,d) were more frequent in mice lacking *Pten* in *Plp1*-expressing cells at baseline and post-DSS recovery when compared to the control and *Pten^fl/fl^;Calb2CreER^T2^* mice. These data are consistent with Kulkarni et al.’s work, where they demonstrated that *Pten* inhibition in *Nestin*-expressing cells exhibited slower transit times; however, our myenteric neuronal cell numbers in the distal ileum are contradictory to their observations [17]. Additionally, we did not see any EdU+Hu+ neurons in any group in our colon sections (Supplementary Figure S1) as is consistent with previous reporting [5,7,34]. These differences could possibly be attributed to the use of different animal strains, intestinal injuries, and methods employed across multiple laboratories.

Odonnel and Puri observed that enteric *PTEN* expression was reduced or absent in patients with Hirschsprung’s disease and intestinal neuronal dysplasia [15]. Our results are consistent, in that *Pten* elimination in our murine models did not protect or improve functional outcomes of DSS-colitis. Be that as it may, we should point out that the absence and/or reduction in *PTEN* in these patient populations occurred during development and the direct cause for variable *PTEN* expression is not yet known. In our model, mice are born healthy and reach maturity with normal levels of *Pten* expression and only then do adult mice undergo tamoxifen-induced *Pten* ablation.

The present work has several limitations which need to be considered. These include but are not confined to (1) that the tamoxifen-inducible cKO mouse models utilized in this study are limited—that is we are unable to directly target enteric *Plp1* and *Calb2* expression. As such, we cannot rule out off target effects from *Pten* elimination in *Plp1*- and *Calb2*-expressing cells throughout the body; (2) only one injury model, DSS-colitis, was tested and our observations may not be applicable to other injury types, such as infectious or ischemic; (3) although we had a satisfactory sample number in all groups, we were only able to inspect two timepoints after DSS-colitis, early (3–4 days) and late (3–4 weeks). We can only speculate if the cellular and functional effects would persist over time.

Our results suggest that conditional *Pten* inhibition in adult glial cells may stimulate gliogenesis. In future studies, it will be interesting to see if *Pten* modification could be useful in diseases or conditions where the intestinal glial population is decreased. In this study *Pten* ablation in adult glia increased the level of neurons and glia in the colon and distal small bowel. It may be worth looking at other injury models and perhaps extended timelines. Finally, as *Pten* modulators have been shown to protect against neuroinflammation [35,36], studies manipulating *Pten* in enteric neuroinflammatory states may also provide promising observations. To conclude, selective *Pten* inhibition in *Plp1*-expressing glial cells presents a valuable approach where increased colonic glial and neuronal numbers and slower intestinal transit times would be advantageous (e.g., short-bowel syndrome and rapid transit disorders) and warrants further studies.

## Figures and Tables

**Table 1:** Animal strains.

Common Name	Strain	Jax Stock No.	RRID * No.
*Pten^flox^*	C;129S4-*Pten^tm1Hwu^*/J	006440	IMSR_JAX:006440
*Plp1CreER^T^*	B6.Cg-Tg(Plp1-cre/ERT)3Pop/J	005975	IMSR_JAX:005975
*Calb2CreER^T2^*	B6(Cg)-*Calb2^tm2.1(cre/ERT2)Zjh^*/J	013730	IMSR_JAX:013730

* Research resource identifier.

**Table 2:** Antibodies.

Antibody	Host	Dilution	Source	Cat. No.	RRID * No.
ANNA-1 (Hu)	Human	1:10,000	Mayo Clinic, Rochester, MN, USA	n/a	AB_2314657
Sox2	Goat	1:100	R&D Systems, San Diego, CA, USA	AF2018	AB_355110
Human Alexa Fluor 488	Donkey	1:400	Jackson ImmunoResearch, West Grove, PA, USA	709-545-149	AB_2340566
Goat Alexa Fluor 647	Donkey	1:400	Invitrogen, San Diego, CA, USA	A21447	AB_2535864

* Research resource identifier.

## Data Availability

The data that supports the findings in this study are available from the corresponding author upon reasonable request.

## Supplementary Materials

The following supporting information can be downloaded at: https://www.mdpi.com/article/10.3390/biom14030346/s1, Figure S1: Hu+ myenteric neurons are not positive for EdU in the colons of mice lacking *Pten* expression in either *Plp1* or *Calb2*-expressing cells.
