# Supplementary material for: Phosphatase and Tensin Homolog Inhibition in Proteolipid Protein 1-Expressing Cells Stimulates Neurogenesis and Gliogenesis in the Postnatal Enteric Nervous System
Source: Biomolecules. 2024 Mar 13;14(3):346. doi: 10.3390/biom14030346 (PMC10967813; doi:10.3390/biom14030346)
Supplement: Supplementary file 1 [file biomolecules-14-00346-s001.zip › Figure S1.pdf]

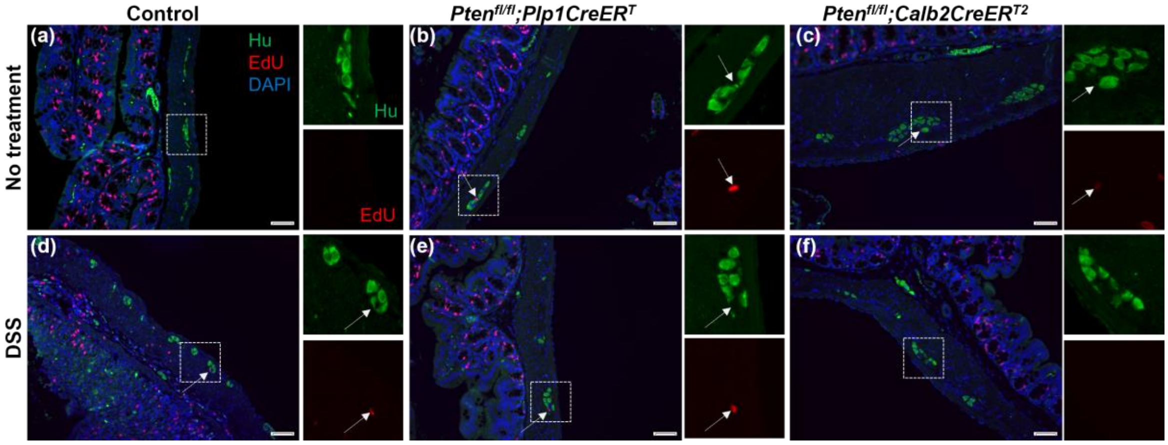

Supplemental Figure S1. Hu+ myenteric neurons are not positive for EdU in the colons of mice lacking *Pten* expression in either *Plp1* or *Calb2*-expressing cells. Representative images of Hu (green) and EdU (red) immunofluorescence on colon sections from control (a, d), *Pten*$^{fl/fl}$;*Plp1CreER*^{T} (b, e) and *Pten*$^{fl/fl}$;*Calb2CreER*^{T2} (c, f,) mice that received no treatment (a, b, c) and 3-4 days post-DSS (d, e, f). Magnified regions are of myenteric ganglia and are indicated with a white square. Arrows are used to highlight EdU+ cells in the myenteric plexus that are negative for Hu; EdU, 5-ethynyl-2'-deoxyuridine; NT, no treatment; scale bar, 50 μm.
